# CLIP: Carbon Dioxide testing suitable for Low power microelectronics and IOT interfaces using Room temperature Ionic Liquid Platform

**DOI:** 10.1038/s41598-020-59525-y

**Published:** 2020-02-13

**Authors:** Ashlesha Bhide, Badrinath Jagannath, Ambalika Tanak, Richard Willis, Shalini Prasad

**Affiliations:** 1 Department of Biomedical Engineering, University of Texas at Dallas, 800W Campbell Rd., Richardson, TX 75080 USA; 2 Department of Electrical and Computer Engineering, University of Texas at Dallas, 800W Campbell Rd., Richardson, TX 75080 USA

**Keywords:** Electrochemistry, Environmental monitoring

## Abstract

Health and safety considerations of room occupants in enclosed spaces is crucial for building management which entails control and stringent monitoring of CO_2_ levels to maintain acceptable air quality standards and improve energy efficiency. Smart building management systems equipped with portable, low-power, non-invasive CO_2_ sensing techniques can predict room occupancy detection based on CO_2_ levels exhaled by humans. In this work, we have demonstrated the development and proof-of-feasibility working of an electrochemical RTIL- based sensor prototype for CO_2_ detection in exhaled human breath. The portability, small form factor, embedded RTIL sensing element, integrability with low-power microelectronic and IOT interfaces makes this CO_2_ sensor prototype a potential application for passive room occupancy monitoring. This prototype exhibits a wide dynamic range of 400–8000 ppm, a short response time of ~10 secs, and a reset time of ~6 secs in comparison to commercial standards. The calibration response of the prototype exhibits an R^2^ of 0.956. With RTIL as the sensing element, we have achieved a sensitivity of 29 pF/ppm towards CO_2_ at ambient environmental conditions and a three times greater selectivity towards CO_2_ in the presence of N_2_ and O_2_. CO_2_ detection is accomplished by quantifying the capacitance modulations arising within the electrical double layer from the RTIL- CO_2_ interactions through AC- based electrochemical impedance spectroscopy and DC- based chronoamperometry.

## Introduction

Monitoring of CO_2_ levels has been a crucial subject of research interest world-wide in regard to efficient building occupancy management for indoor occupancy comfort and energy-savings^1^. In the US, indoor air quality monitoring and occupancy comforts account for 40% of the total energy usage^2^. Intelligent buildings have adopted system controls that communicate with the deployed sensor network within the building to optimize occupancy comforts and energy consumption. IOT based sensor technology is gaining attraction and has made its way into building management systems to monitor vital indoor environmental parameters such as acoustics, CO, VOC, small particulate matter, CO_2_, temperature, and humidity. Information collected from all these sensors can be utilized to predict patterns for preventing mishaps and take corrective actions in advance for effective building maintenance^3^. The smart sensor network should be capable of automatically modulating its air ventilation to avoid excessive ventilation for energy savings in areas with highly variable and dense occupancy^4^. Exhaled human breath is the main source of CO_2_ production in indoor spaces and is a widely used indicator of room occupancy. CO_2_ is regarded as a toxic contaminant with acceptable exposure limit of 5000 ppm over an 8-hour window or a short exposure limit of 15,000–30,000 ppm for 15-minutes according to OSHA and ASHREA standards. The CO_2_ levels produced by humans are much higher than the CO_2_ present in outdoor environment. Studies show that the indoor CO_2_ concentration is 700 parts per million (ppm) higher than the concentration of CO_2_ (~450 ppm) in the outdoor environment per person (ASHRAE standards). Although, there is a latency observed in CO_2_ concentration equilibration for accurate estimation of ventilation rates from CO_2_ levels due to timely variation in room occupancy and ventilation rates; CO_2_ concentration can still be regarded as a proxy standard to measure ventilation per person^4^. Typically, 350–1000 ppm is an acceptable indoor CO_2_ level in spaces with good ventilation. Exposure to CO_2_ levels >1000 ppm is considered as a hazard affecting human cognition, loss of consciousness, and causes permanent heart damage making it a relevant detecting element for diagnosing physical conditions. CO_2_ monitoring also finds its use in a multitude of applications such as automobile industries, medical facilities, breweries, greenhouses etc. Hence, a practical solution is required to protect the population and maintain a safe environment. Integration of low- power microelectronics with sensing platforms offer a solution for room occupancy monitoring by providing real-time gas analysis of indoor CO_2_ with minimal user interference. Such a device needs to be portable, handheld, low-cost, low-power, small-size, sensitive to CO_2_, and highly selective to CO_2_ over other environmental gases and volatile organic compounds (VOC). The current CO_2_ sensing technologies are based on non-dispersive infra-red spectroscopy (NDIR), nanotexturing of metal oxide films, and photoelectrochemical techniques^5,6^. Although NDIR sensors are robust, they require long stabilization times, consume high power, and have a response time in the order of minutes^7,8^. Metal-oxide sensors require high operational temperatures to break down gases into reactive species for interactions to occur with the sensing films; they are cross-sensitive to interferent gases and suffer from purity issues during manufacturing^9,10^. Although small form factors can be achieved for electrochemical sensors, they have a short life- time from continuous gas exposure, response time is dependent on the time required by the gas to diffuse through the barrier, narrow operational temperature range, and cross-sensitivity issues^11^. Application and development of new material systems can be a path to circumvent the disadvantages of the current sensing technologies. Room temperature ionic liquids (RTIL) are a new class of materials that can be utilized as a low-power, easy maintenance solution to develop a portable gas sensing system. RTIL’s are solvent free electrolytes consisting of cation/anion pairs. RTILs possess characteristic properties such as high ionicity, low volatility, high physical and chemical stability, and wide electrochemical window which are advantageous from the perspective of gas sensing applications^12^. Our group has previously characterized various RTILs and demonstrated their feasibility for CO_2_ and humidity sensing^13–16^.

In this paper, we have described the functionality and utility of a developed low-power microelectronic research prototype integrated with an RTIL-based electrochemical sensing platform for the detection of CO_2_ in exhaled breath as a measure of room occupancy. The performance of this research prototype was benchmarked against a commercial standard Vaisala CO_2_ sensor. We have provided a comprehensive performance characterization of the RTIL EMIM[TF_2_N] as a suitable sensing element for the development of a robust electrochemical CO_2_ sensor. In our study, we have leveraged the electrochemical double layer formation of the RTIL moieties to capture the interfacial modulations occurring as a result of RTIL-CO_2_ interactions at the RTIL- electrode interface through electrochemical impedance spectroscopy and chronoamperometric techniques.

## Results and Discussions

The organization of this section organized is as follows: (1) Rationale behind using the interdigitated electrode design (2) EMIM[TF_2_N] as a suitable candidate for CO_2_ sensing (3) Mechanism of EMIM[TF_2_N] -CO_2_ interaction for electrochemical gas sensing (4) Characterizing the CO_2_ binding interaction with EMIM[TF_2_N] for electrochemical gas sensing (5) Translatability of the RTIL- CO_2_ interaction towards low power portable microelectronic prototype development (6) Development and evaluation of the low power portable prototype in real-time environment for CO_2_ detection (7) Validation of the low power portable prototype for real-time CO_2_ detection.

### Rationale behind using the interdigitated electrode design

**Figure 1:**
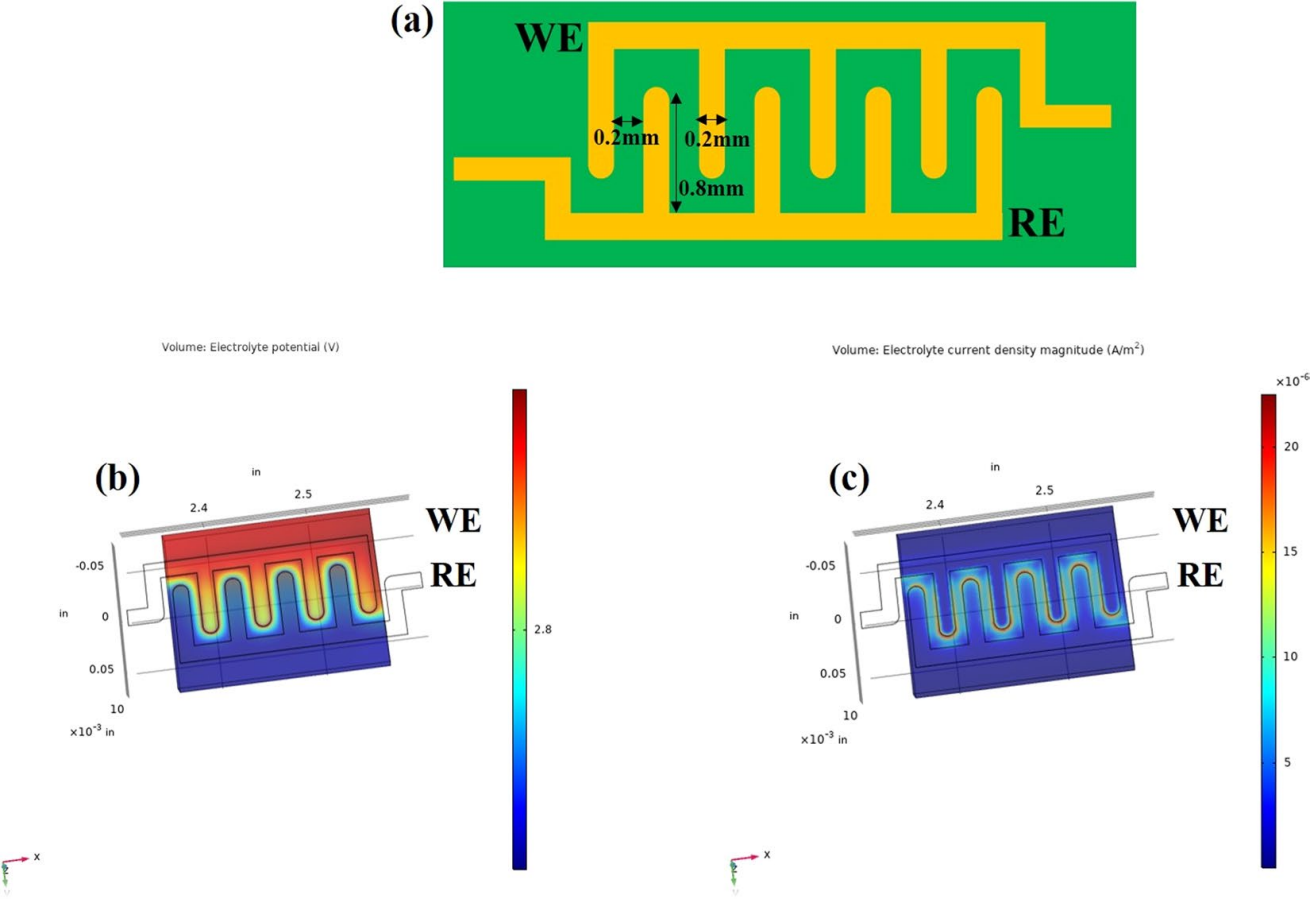
(**a**) Schematic of the interdigitated electrode sensor design for CO_2_ sensing (**b**) COMSOL simulation representing electrolyte potential for working and reference electrodes (**c**) COMSOL simulation representing electrolyte current density for working and reference electrodes.

Interdigitated electrode designs (IDE), as shown in Fig. 1a, have been used commonly to build planar capacitive sensor that allow for easy integration on low power electronic platforms^17,18^. The increased surface area of IDE’s produce amplified output signals from the higher electrical fields confined within smaller geometries. IDE’s are advantageous due to the ease of fabrication process, enhanced sensitivity, enhanced detection limits, and allows operation with low sample volumes. The IDE’s measure the change in the dielectric permittivity of RTIL upon interaction with gas. Gold is a suitable candidate material for IDE’s due to its stable electrochemical properties and chemical inertness. COMSOL simulations were performed to simulate the behavior of the gold IDE’s in the presence of EMIM[TF_2_N] as the electrolyte in real operating environment. Electrical properties of gold are applied to both the working (WE) and the reference electrodes (RE). A constant AC potential of 10 mV with a DC bias of 2.8 V (optimized electrochemical voltage of EMIM[TF_2_N]) is applied to the WE with respect to RE to obtain enhanced sensing performance; the RE is grounded/insulated. The electric fields are confined within the RTIL- electrode interface boundaries. The electrolyte potential distribution and the electrolyte surface current density plots from the one digit of the WE to the adjacent digit of RE at the RTIL- electrode interface is shown in Fig. 1b,c respectively. The electrolyte potential is maximum at the digits of the WE and varies from 2.8 V to 0 V moving from WE to the RE. Maximum current density is observed at the WE which influences the output response of the system. The surface current densities contributed by the electrolyte from WE to RE decreases from 33 μA/m^2^ to 0 µA/m^2^ and no parasitic currents are contributed from the electrodes going from WE to RE. The group has previously demonstrated enhanced CO_2_ sensing performance on gold linear IDE design vis-à-vis carbon circular IDE design^13^. The equations governing the defined boundary conditions, simulated potential distribution (see Fig. S1a), and simulated current densities (see Fig. S1b) are listed in the supplementary section [S1].

### EMIM[TF_2_N] as a suitable candidate for CO_2_ sensing

1-Ethyl-3-methylimidazolium bis(trifluoromethylsulfonyl)imide (EMIM[TF_2_N]) is an easily available commercial RTIL with excellent electrochemical properties that can be utilized for CO_2_ sensing^19–22^. Literature studies reveal that RTIL’s with fluorinated anions have large CO_2_ absorption capacity. TF_2_N^−^ has shown to have highest solubility in CO_2_ in comparison to other fluorinated anions PF^−^_6_ and BF^−^_4_^13,23,24^. Weak Lewis acid-base interactions were observed between the fluorinated anion and CO_2_ wherein the anion acts as the acid. Cyclic voltammetry studies have shown that increasing levels of CO_2_ produced increased cathodic peak currents due to increased production of O_2_^−^ radicals indicating the uptake of CO_2_ by EMIM[TF_2_N]. A decreased peak current is observed in the reverse scan indicating an irreversible reaction between O_2_^−^ radicals and CO_2_^25^. Desorption of CO_2_ requires subjecting the RTIL to thermal treatment. A combination of imidazolium salts with TF_2_N^−^ have been used for CO_2_ sequestration primarily due to its low viscosity, hydrophobicity, and inertness to moisture. Decreased length of the cationic alkyl chain is beneficial as it decreases the interfacial tension of the RTIL with moisture and exhibits greater ionic conductivity than longer cationic alkyl chain RTIL’s^26^. EMIM[TF_2_N] has shown greater selectivity to CO_2_ than other gases present in the atmosphere owing to its lower Henry’s constant that dictates the capacity of gas uptake by an RTIL. EMIM[TF_2_N] has a Henry’s constant of 3.96 which allows it to specifically interact with CO_2_ in comparison to other gases present in ambient environment that have a Henry’s constant almost 10x greater than that of CO_2_^19,27^.

### Mechanism of EMIM[TF_2_N] - CO_2_ interaction for electrochemical gas sensing

**Figure 2:**
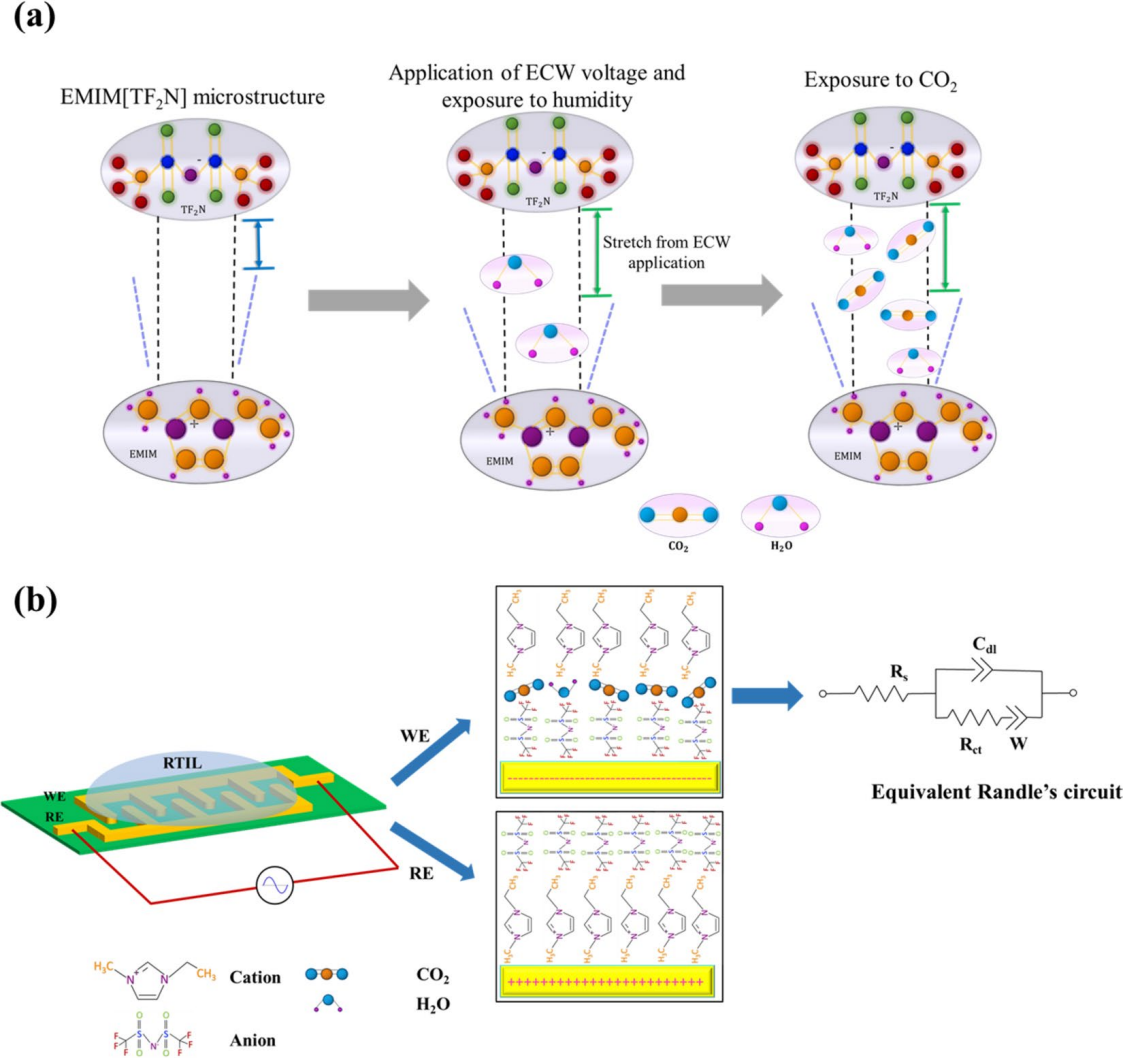
(**a**) Schematic of interaction of EMIM[TF_2_N] with CO_2_ on application of ECW voltage under test temperature and humidity conditions. (**b**) Schematic representing the electrical double layer formation at the RTIL- electrode interface.

Ionic liquid-based systems are highly complex electrochemical systems and a model of electrode- ionic liquid interface and the potential distribution with the double layer as not been well-defined yet. However, the potential difference across the electrified electrode surface and the ions in the bulk solution result in the formation of a capacitive electrical double layer (EDL)^28^. IL’s are subjected to a variety of forces – Coulombic, Van der Waal’s, and hydrogen bonding^29,30^. These forces lead to a structured arrangement of ions at the IL interface due to the clustering of similar molecular groups. Studies suggest that IL interface are composed to three layers: an innermost interfacial of counterions are electrically absorbed on the electrified interface, a bulk liquid region, and a transition region wherein the electric potential decays from the innermost region to the bulk region^31^. The interfacial layer is highly organized with the alternating cation- anion pair structures electrostatically bound to each other extending into the transition zone. The hypothesis presented is that application of potential, temperature, and humidity stretches the bonds between that cation-anion pairs creating interstices providing room for the CO_2_ molecules to dock into the gaps and interact with TF_2_N^−^ anions of the RTIL^14,32^ as shown in Fig. 2a. The interactions occurring at the RTIL- electrode interface have been probed by capacitance measurements obtained from electrochemical impedance spectroscopy and chronoamperometry. Electrochemical impedance spectroscopy is an AC based- dielectric spectroscopic technique which characterizes the physical and the chemical events occurring within the EDL through the application of a small AC perturbation voltage with a DC bias. The impedance of the system is computed by measuring the change in current caused by the change in dielectric behavior of the system. The RTIL- electrode interface can be represented as a modified Randle’s circuit as shown in Fig. 2b constituting of a parallel RC element in series with a diffusion driven constant phase element called the Warburg impedance^33^. The interaction of RTIL -CO_2_ modulates the double layer capacitance denoted by C_dl_; the R_ct_ component accounts for any charge transfer occurring between the RTIL and the electrode surface. Dominant C_dl_ effects have been observed in frequency regime 100–1000 Hz. We chose a calibration frequency of 100 Hz to represent the system’s response as a capacitive phase is observed at that frequency [See supplementary information S2]. Chronoamperometry (CA) is a DC-based signal transduction technique that captures the charge-discharge dynamics of the EDL. The current response (I α e^−t/R^_ct_^C^_dl_) of the system as a function of the charge- discharge is captured with respect to time^34^.

The CO_2_- RTIL interactions occur primarily through Van der Waal’s interaction between TF_2_N^−^ anion and CO_2_. Cationic chain length plays a secondary role in increasing the solubility of CO_2_ in the RTIL^35^. The applied DC bias of the system depends on the optimal electrochemical window voltage (ECW) of the RTIL at which the CO_2_ sensing response is maximum. At optimal ECW voltage, increased interactions occur between CO_2_ and the higher anionic densities of TF_2_N^−^ at the electrode surface resulting in increased capacitance responses of the system. The RTIL- CO_2_ interaction mechanism probed by EIS and CA techniques allows for the development of a noninvasive rapid gas sensing technology.

### Characterizing the CO_2_ binding interaction with EMIM[TF_2_N] for electrochemical gas sensing

**Figure 3:**
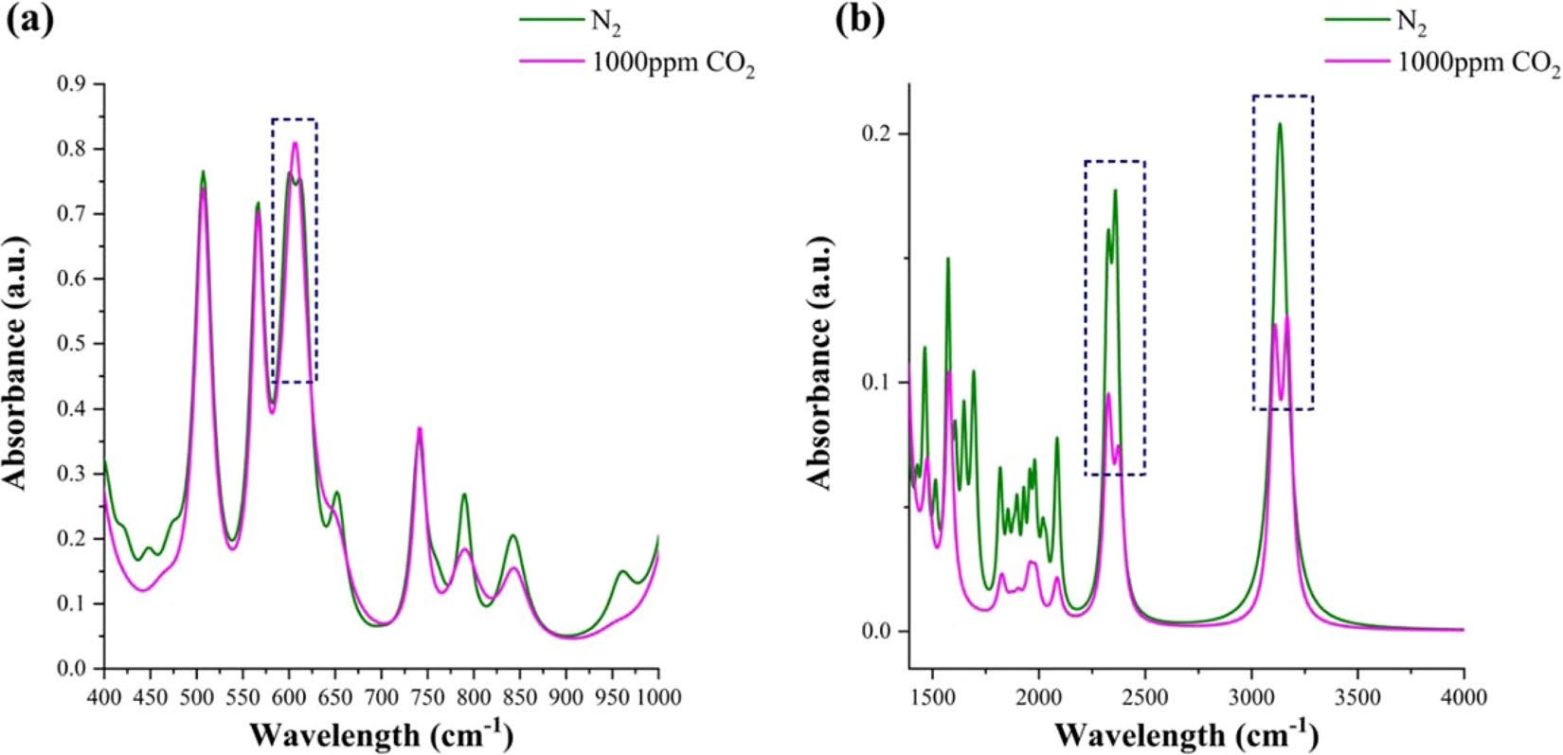
FTIR spectrum of RTIL -CO_2_ interaction. Boxed regions represent the spectral peak changes on RTIL -CO_2_ interaction (**a**) at ~660cm-1 (**b**) at ~2330, 2363, 3100, and 3150 cm^−1^.

While EIS and CA unravel the events occurring at the interface level as a function of frequency and time respectively, the binding interaction of EMIM[TF_2_N] with CO_2_ can be studied through Fourier transform IR spectroscopy (FTIR). FTIR spectral analysis allows identification of molecular moieties and structures through absorption bands. In this study, information obtained from the FTIR analysis is used to confirm the EMIM[TF_2_N] -CO_2_ interactions responsible for the electrochemical detection of CO_2_. FTIR has been used to distinguish between the spectral components of the RTIL prior to and after interaction with CO_2_ through spectral deconvolution. Figure 3a,b shows the deconvoluted FTIR spectrum for EMIM[TF_2_N] exposed to N_2_ and 1000 ppm CO_2_ respectively. The active bending of CO_2_ is represented by the appearance of a shoulder peak at ~660 cm^−1^ (Fig. 3a). The resolved peaks appearing at ~2330 cm^−1^ and 2363 cm^−1^ represent the anti-symmetric mode of CO_2_ stretching on interaction with the fluorinated moiety of the RTIL (Fig. 3b). The peaks appearing at 3100 cm^−1^ and 3150 cm^−1^ (Fig. 3b) can be attributed to the adsorption doublet band because of CO_2_ stretching^36^. The results obtained from the FTIR spectral analysis confirm the interaction of the CO_2_ with fluorinated moiety of the RTIL further making the use of RTIL’s as a promising sensing element for integration on portable microelectronic and IOT interface platforms for the detection of CO_2_ in environmental applications.

### Translatability of the RTIL- CO_2_ interaction towards low power portable microelectronic prototype development

#### Evaluation of sensor operation conditions for optimal CO_2_ sensing performance

Effect of varying operating voltage bias conditions on CO_2_ sensing response. To understand the effect of DC bias voltages on the CO_2_ sensing response of the system, we chose three DC voltages within the ECW − 1.6 V, 2.3 V, and 2.8 V. The capacitance responses at all three ECW voltages were captured at 400,750, and 1000 ppm CO_2_ concentrations under varying environmental temperature and humidity conditions: 25 °C 25% RH, 45 °C 45% RH, and 65 °C 65% RH. The capacitance change at every CO_2_ concentration under varying test temperature and humidity conditions is computed as given below-

*Capacitance change at 100* *Hz (pF)* = *Capacitance captured for N*_2_ *baseline at given temperature and humidity at 100* *Hz (pF) - Capacitance captured for a particular CO*_2_ *concentration at given temperature and humidity at 100* *Hz (pF)*.

**Figure 4:**
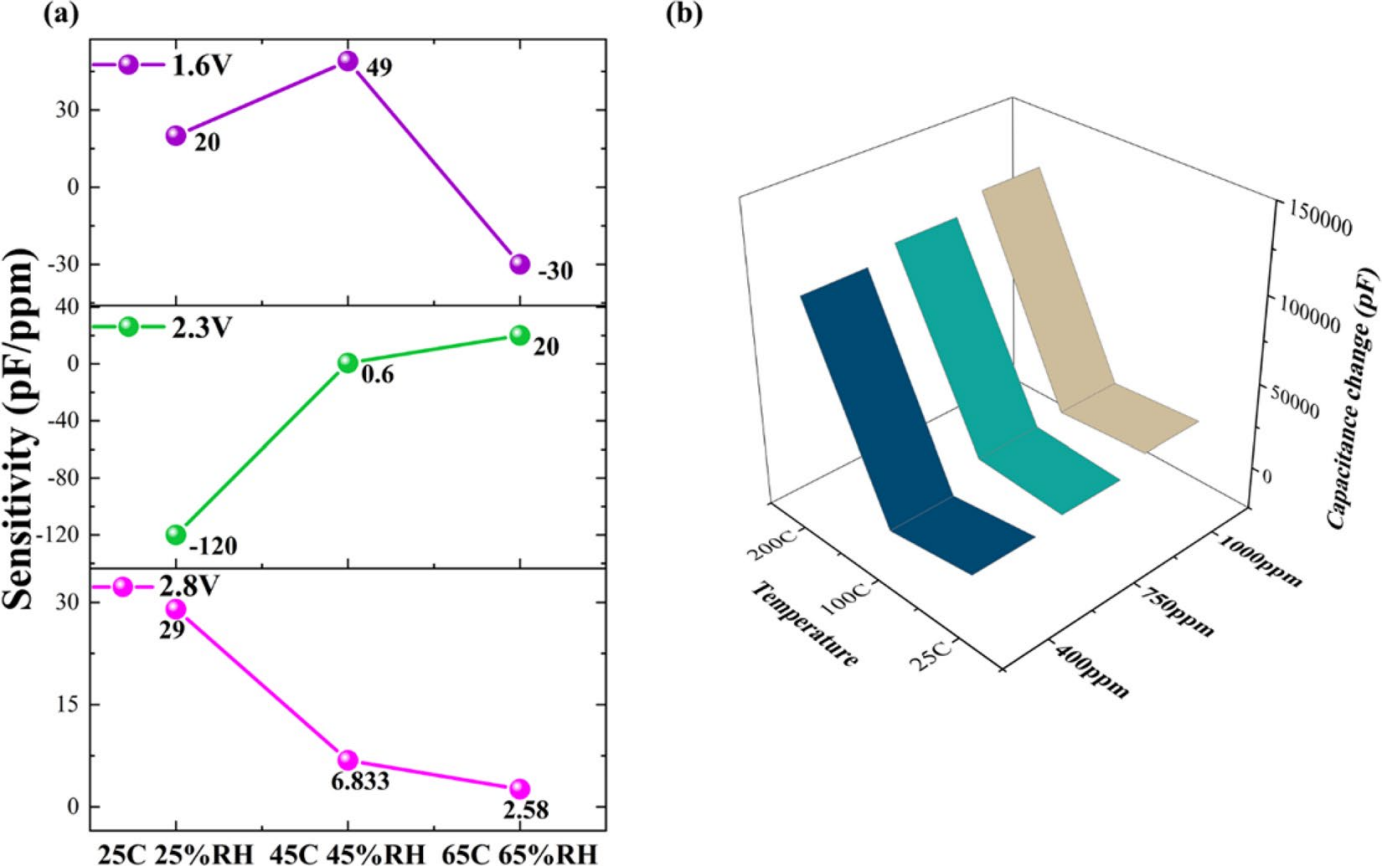
(**a**) Sensitivity performance of EMIM[TF_2_N] across ECW voltages – 1.6, 2.3, 2.8 V for varying temperature and humidity conditions. (**b**) Temperature response of EMIM[TF_2_N] at ambient and elevated conditions.

At 1.6 V, the trend in capacitance change is linear with increasing temperature and humidity at 400 ppm CO_2_. However, it inverts at 65 °C 65% RH for 750 and 1000 ppm CO_2_ concentrations as shown in Fig. 4a. At 2.3 V, a decrease in capacitance change is seen going from 25 °C 25% RH to 45 °C 45% RH with a sudden increase in capacitance change at 65 °C 65% RH. The ECW voltages 1.6 and 2.3 V, no definitive relationship between capacitance change slopes and increasing CO_2_ concentrations is observed. However, at 2.8 V, a monotonically increasing slope in capacitance change is observed with increasing CO_2_ concentrations. Additionally, the capacitance change is seen to be decreasing with increasing temperature and humidity conditions at 2.8 V. This behavior can be attributed to the enhanced polarization occurring at higher DC bias potential leading the EDL into an overcrowded state that can be leveraged for enhanced CO_2_ sensing response. Overall, a 15% higher capacitive response towards CO_2_ sensing is observed at the maximum bias voltage of 2.8 V than 1.6 V.

The sensitivity of EMIM[TF_2_N] to CO_2_ concentrations across 400–1000 ppm at 1.6, 2.3, and 2.8 V is shown in Fig. 4a. The sensitivity of EMIM[TF_2_N] at 1.6 V is observed to be 20, 49, −30 pF/ppm with increasing temperature and humidity. Positive sensitivity is observed at 25 °C 25% RH and 45 °C 45% RH, but it switches to negative at 65 °C 65% RH. At 2.3 V, negative sensitivity of −120 pF/ppm is obtained at 25 °C 25% RH which flips at 45 °C 45% RH and 65 °C 65% RH. At the edge of the ECW voltage of 2.8 V, a positive decreasing trend in sensitivity from 29–2.58 pF/ppm is observed across increasing temperature and humidity conditions. The sensor on operation at 2.8 V shows no trend reversals in its capacitive and sensitivity responses deeming it to be the optimal DC bias voltage for CO_2_ sensing.

Sensor operation at the edge of electrochemical window voltage. The DC bias voltage applied to the RTIL-electrode interface greatly influences the signal response pertained to CO_2_ sensing at varying temperature and humidity conditions. The RTIL on contacting the surface of the electrode forms a thin-multilayer EDL of cation-anion pairs due to the inherent charge on the electrode surface. The charge density in the EDL can be modulated to augment the output response by applying higher DC voltage bias with the electrochemical window of the RTIL’s operation. The ECW voltages for EMIM[TF_2_N] lie within the range −2.0– + 2.9V^37^. Beyond this range, the structure and thus the performance of the RTIL is diminished. In solvent-free ionic electrolyte systems such as RTIL the density of charges in the EDL may exist in either of the two states – Overscreened or Overcrowded^38^. Overscreening effect is observed at small voltages where more countercharges tend to neutralize the surface charges at the EDL interface thereby screening the potential from the next layer of charges. However, at higher DC bias voltages overcrowding is observed wherein a concentrated density of charges build up in the EDL allowing for an increase in the output capacitive response of the system on exposure to increasing CO_2_ concentrations.

Effect of elevated temperature on the on CO_2_ sensing response. For high temperature applications such as in automobiles, it is critical to understand the effect elevated temperatures have on the CO_2_ sensing mechanism and performance. The evaluation of CO_2_ sensing performance at increasing CO_2_ concentrations 400, 750, and 1000 ppm at 25, 100, and 200 °C with 0% humidity is shown in Fig. 4b. At 25 °C, an increasing response in capacitance change is observed from 7000–18000 pF Similar trends in capacitance changes are observed at 100 and 200 °C with increasing CO_2_ concentrations due to enhanced C_dl_ effects. At 200 °C, the change in capacitance across 400–1000 ppm CO_2_ is observed to be 10600–12200 pF. The greater magnitude of response obtained at 200 °C can be hypothesized to be due to the weakening of intermolecular bonds between cation-anion pairs allowing for more CO_2_ molecules to dock within the interstices thus modulating the C_dl_. An overall RSD of <2% is observed.

#### Evaluation of sensor performance metrics for optimal CO_2_ sensing performance

The optimization of certain static and dynamic characteristics is necessary for the calibration of performance metrics of a sensor^39^. We have utilized characteristics such as sensitivity, selectivity, repeatability, dynamic range of detection, and hysteresis at ambient conditions to represent the performance of the developed RTIL- based gas sensor.

Sensitivity is defined as the slope of the calibration curve plotted across CO_2_ concentrations 400–1000 ppm at varying test temperature and humidity conditions. At the optimal ECW voltage of 2.8 V, maximum sensitivity of 29 pF/ppm is obtained at ambient temperature and 25% RH which drops down with increasing temperature and humidity conditions as shown in Fig. 4a. Similar CO_2_ sensitivity was obtained as reported previously^40,41^.

**Figure 5:**
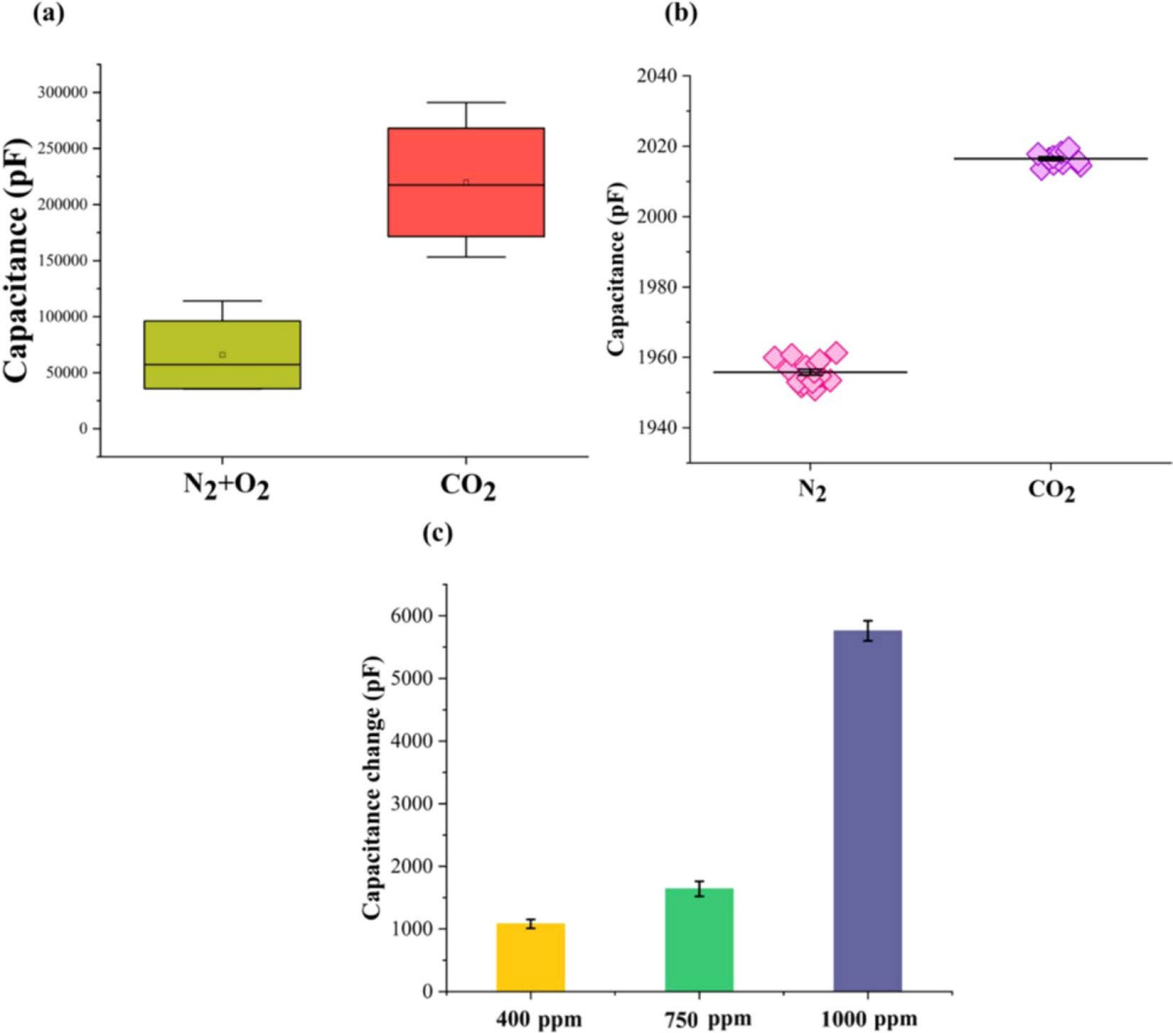
(**a**) Selectivity performance of EMIM[TF_2_N] in detecting CO_2_ in the presence of atmospheric interferents N_2_ and O_2_. (**b**) Repeatability performance of EMIM[TF_2_N] in detecting CO_2_ over multiple measurements (N = 20). (**c**) Continuous cycling performance of EMIM[TF_2_N] over multiple cycles (N = 5).

The reliability of the sensor depends on two parameters: Selectivity and repeatability. Selectivity refers to the ability of the RTIL sensor to respond specifically to a particular gas i.e. CO_2_ over other environmental components such N_2_ and O_2_ which are present abundantly in the environment. The selectivity performance of 1000 ppm CO_2_ concentration over N_2_ at ambient temperature and 25% RH is shown Fig. 5a. The developed sensor shows a distinguishable capacitance response in detecting the non-specific gases (N_2_ + O_2_) over specific CO_2_ gas. The average signal obtained from the non-specific gases (N_2_ + O_2_) is 65000 pF while the average signal obtained from CO_2_ interaction is 210000 pF which is estimated to be a ~3 times larger signal than the non-specific signal. The detection limit can be identified to be 400 ppm CO_2_ and the dynamic range of the sensor is 400–1000 ppm.

Repeatability of a sensor is a measure of overall drift or deviation in the N_2_ baseline and the CO_2_ sensing response over a period of time. The repeatability in the capacitive responses of the RTIL sensor in measuring N_2_ and CO_2_ responses alternatively over n = 20 measurements at ambient temperature and 25% RH is shown in Fig. 5b. This performance characteristic would give an understanding of the natural adsorption- desorption dynamics of the RTIL at ambient temperature. The sensing platform recovers to a similar baseline after desorption. The capacitance of the N_2_ baseline lies at 1955 ± 4 pF and the magnitude of capacitance response for CO_2_ baseline is measured to be 2016 ± 3 pF. The distinguishability and minimal variances in the cycling responses over a 4-hour duration for N_2_ and CO_2_ makes this RTIL platform suitable for reliable, selective detection of CO_2_ in ambient environment.

The continuous cycling performance of the sensor for N_2_ and CO_2_ over 5 cycles at ambient temperature and 25% RH is shown in Fig. 5c. The RTIL sensing element is subjected to thermal treatment after every CO_2_ cycle to desorb the CO_2_ gas completely and allow for a baseline reset. The capacitance changes computed from the N_2_ baseline to CO_2_ concentrations − 400, 750, 1000 ppm for each cycle are found to be 1080 pF (RSD ± 0.7%), 1640 pF (RSD ± 0.12%), and 5760 pF (RSD ± 0.16%) respectively. A concentration dose dependent response is observed over 5 cycles and the % RSD variation can be attributed to the influence of humidity on the fluorinated anion- CO_2_ interaction. The sensor performance metrics for elevated conditions −65 °C and 65% RH for varying CO_2_ concentrations are shown in supplementary section [Refer S3].

### Development and evaluation of the low power portable prototype in real-time environment for CO_2_ detection

A portable CO_2_ sensing prototype using EMIM[TF_2_N] as the sensing element interfaced with low-power MSP 430 has been developed for human use.

**Figure 6:**
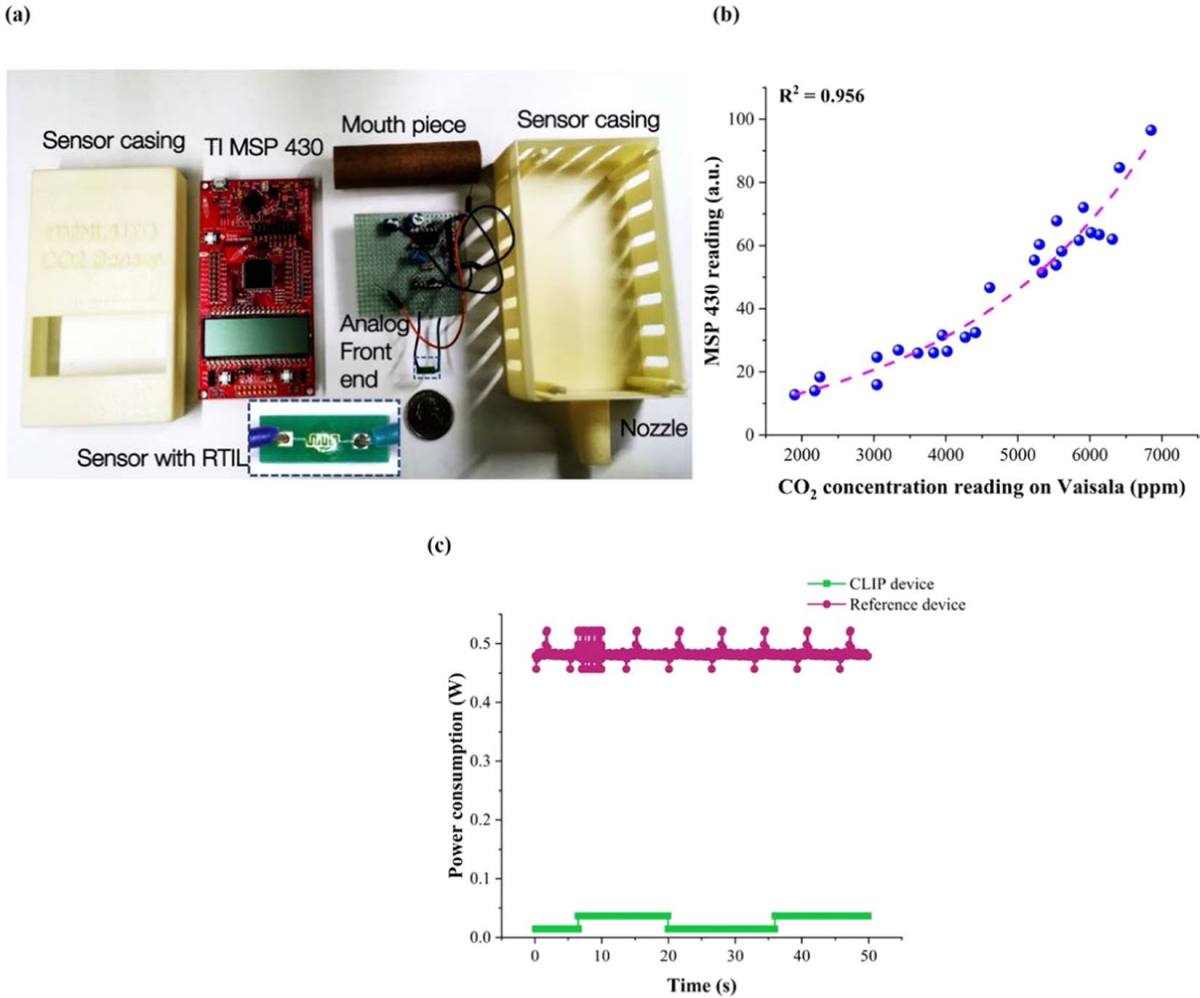
(**a**) Picture representing the CO_2_ sensor research prototype comprising of RTIL coated IDE, analog front end constituting of electronic circuitry, and a MSP 430 microcontroller. (**b**) Calibration curve established for the CO_2_ sensor with respect to commercial device Vaisala. (**c**) Comparison of power consumption between CLIP device and reference device Vaisala.

The prototype device, as shown in Fig. 6a, is a three-stage device consisting of (1) IDE sensing electrodes coated with RTIL (2) An electronic circuit to measure and amplify the chronoamperometric current output obtained from the RTIL-coated IDE in response to the CO_2_ concentration being measured (3) TI MSP 430 microcontroller with a processing algorithm that displays a reading of CO_2_ concentration (ppm) that the sensing platform was exposed to. The electronic circuit consists of voltage divider circuit to step down the battery power supply to the ECW voltage (2.8 V) required for the operation of the RTIL sensing platform. The real-time output current is then fed into a variable gain amplifier to obtain an amplified real- time current which is further processed by the MSP 430. The sensor algorithm running on the MSP 430 microcontroller measures the current through the shunt resistor over time and reports the difference between the time average and a baseline figure obtained from the ambient environment. Furthermore, a calibration curve is developed to convert this average to a CO_2_ concentration. This calibration curve is obtained for a modulated flow of a pre-mixed CO_2_ concentration on the RTIL sensor and the Vaisala sensor (commercial device) at the same time to ensure the same CO_2_ concentration reaches both the sensors. The maximum value shown by each sensor is recorded and displayed in Fig. 6b. The resulting relationship is used to develop a calibration curve with an R^2^ correlation coefficient of 0.956. The resulting equation is inverted and programmed into the MSP 430 microcontroller to display a CO_2_ concentration (See supplementary video SV1). The developed sensing platform is capable of demonstrating a dynamic range from 400–8000 ppm with a short response time of ~10 s. The dynamic range encompasses the ambient and breath CO_2_ ranges matched to the CO_2_ concentration range that the Vaisala can detect.

This prototype requires a very simplistic analog front end due to the high signal output obtained from the RTIL sensing platform. MSP 430 from Texas Instruments is a low-power microcontroller,

that was leveraged to develop the portable CO_2_ monitor. Low-power of the device was validated and compared against the standard device. Instantaneous power consumption of the device was compared with the standard reference device. The two reference techniques discussed in this work is NDIR (full form) and chronoamperometry. NDIR is dependent on physical phenomena of light diffraction, hence a light source is incident on the gas sample. The dispersed light is sensed using an optical detector and converter to a current value. Thus, the outcome of an NDIR measurement depends on the number of molecules present in the gas sample at the instance of measurement. Thus, the measurement happens only when the device is active. Chronoamperometry is an electrochemical sensing technique which relies on molecular binding of CO_2_ at the sensor surface. Thus, the sensor is capable of measuring a gas sample without being excited. The voltage excitation is applied only when a measurement is desired from the sensor, in turn saving power requirements.

The average power consumption of the system can be calculated using the product of supplied voltage and current over a fixed period T. Since the supplied voltage is constant, we will focus our analysis on the changes in current.

$${\rm{P}}={\rm{VI}}$$

$$q={\int }^{}i\cdot dt$$

Since the charge of the system is conserved, meaning, a battery source can provide only a fixed amount of charge, the current flow and the time of flow are inversely proportional.

$$i\,\alpha \frac{1}{{\rm{d}}t}\Rightarrow \int ia\int \frac{1}{dt}\,\Rightarrow \,I\alpha \frac{1}{T}$$

The above relationship can be applied to the current measurements done on the reference device and the CLIP device by integrating the measured current waveform.

In case of the reference device, the current consumption is maximally constant for a given time T, leading to a constant current consumption of a certain value I_peak_. This leads to a constant power consumption for this device.

In case of the CLIP device, the measurement is done periodically at T = 20 s. Here, the device is actively measuring for a period of T_on_ = 10 seconds, whereas is sleeps for the rest. In such a case, the average current flow will depend on the different current values I_active_ and I_sleep_ and their respective periodicity.

$$I=\{{I}_{active}\frac{{T}_{on}}{T}\}+\{{I}_{sleep}\frac{{T}_{off}}{T}\}where\,T={T}_{on}+{T}_{off}$$

In the above relationship, the value of I_active_ and I_sleep_ are suppressed due to the ratio of T_on_ and T_off_, thus, the average current value is reduced significantly. Thus, less power is consumed in the proposed CLIP device. Figure 6c demonstrates the power consumption of the entire system. The developed CLIP device consumes a maximum power of 36 mW in comparison to the reference device of ~480 mW. The power consumption of this is at least lower by 10 times lower in magnitude when compared to the standard reference device. Furthermore, the device has lower power consumption than the CO_2_ monitor reported in [40]. This validates the low-power consumption of the device reported in this work.

### Validation of the low power portable prototype for real-time CO_2_ detection

The performance of the developed prototype CO_2_ sensor is compared and benchmarked against a commercial CO_2_ sensor- Vaisala by utilizing two statistical techniques: (1) Regression analysis to establish a calibration between the developed sensor and the commercial sensor as described in the above section (2) Bland-Altman analysis to quantify the agreement in the responses obtained from the developed sensor and the commercial sensor.

**Figure 7:**
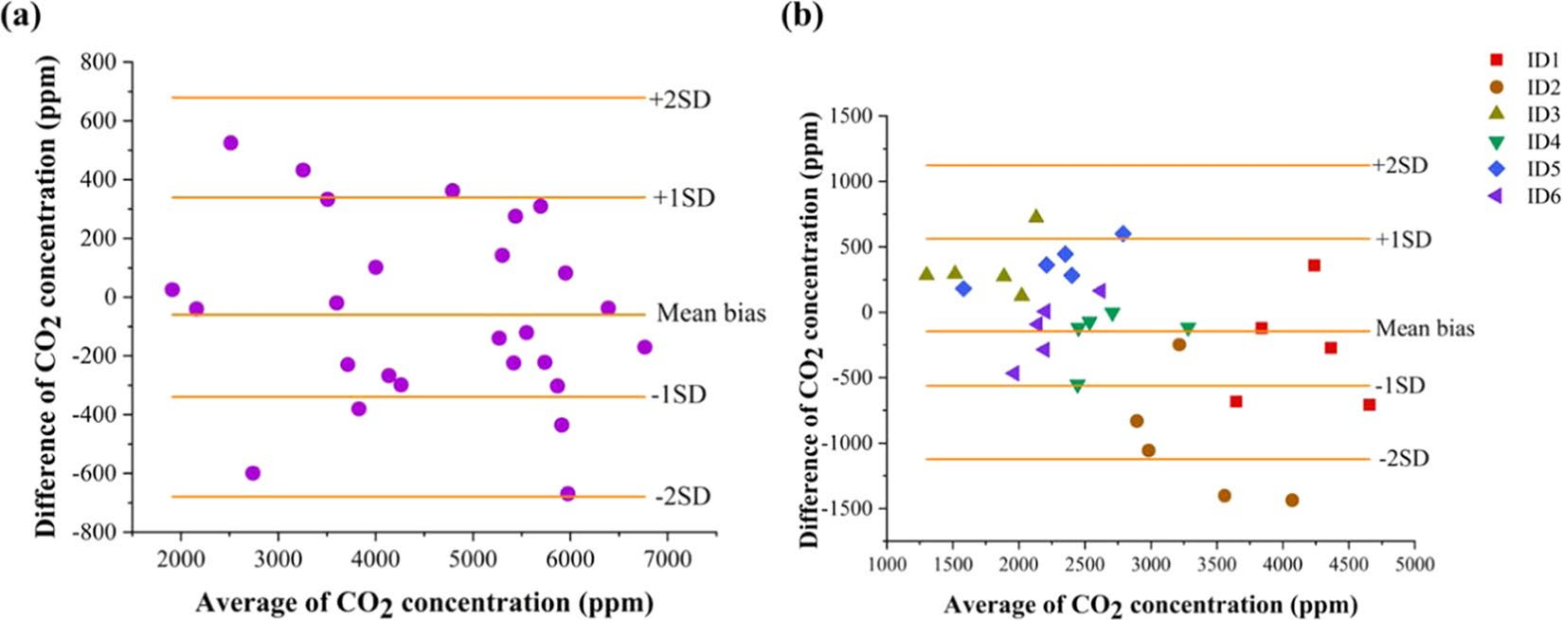
(**a**) Comparison of the developed research prototype with commercial sensor Vaisala for CO_2_ concentrations 2000–7000 ppm. (**b**) Comparison of the developed research prototype with commercial sensor Vaisala for exhaled breath CO_2_ concentrations from six human subjects.

Bland-Altman analysis is performed to analyze the relationship between the CO_2_ concentrations measured by both the prototype device and commercial device and is shown in Fig. 7a. The mean bias is −60.2 ppm which lies close to zero and the ±1 SD of the bias is ±339.5 ppm. All measurements except one fall within ±2 standard deviations but most of the measurements fall within one standard deviation. From the aspect of real-time application, the performance of both the sensors were compared by measuring the breath concentrations of six human subjects (three male and three female subjects). The participants exhaled (same breath) into both the sensors simultaneously and as consistently as possible. Three minutes elapsed in between measurements to allow both sensors to equilibrate. Data was collected in accordance with the relevant guidelines and regulations through approval by the University of Texas at Dallas Institutional Review Board. Informed consent was obtained from all participants (all participants 18 years or older) before data collection. The Bland-Altman analysis obtained for the human subjects study is shown in Fig. 7b. The mean bias is observed to be −145.43 ppm and the ±1 SD of the bias is ±561.2 ppm. The differences between the Vaisala and the RTIL sensor are also evenly distributed about a difference of zero. All but two measurements fell within two standard deviations and most fell within one standard deviation demonstrating the viability of the RTIL based sensor. The large deviation in the statistics are due to the subject to subject variations in breathing patterns.

## Conclusion

This work is a first-time demonstration of a portable, low-power microelectronic platform for rapid and dynamic detection of CO_2_ in exhaled breath making it a suitable device for use in indoor air quality monitoring for room occupancy applications. An empirical approach towards utilization of a unique sensing element – RTIL is evaluated towards development of a low-power CO_2_ gas. The fluorination of the anionic moiety of the ionic liquid EMIM[TF_2_N] benefits our application from its greater affinity to capture CO_2_ selectively over other gases in ambient atmosphere. Gold linear IDE’s known for their increased surface are allows for enhanced signal response. The COMSOL results display the effect of geometry and electrode design in validating the enhancement of the CO_2_ signal response. The increased Vander Waal’s interactions between the fluorinated anion and CO_2_ at the optimal electrochemical voltage is leveraged to obtain maximum output signal response. Electrochemical impedance spectroscopy and chronoamperometry techniques are utilized to capture the RTIL- CO_2_ interaction events occurring at the interface of the double layer. FTIR studies confirm the RTIL-CO_2_ interactions through spectral peak changes and wavelength shifts. ECW optimization reveals maximum capacitance response at 2.8 V. A temperature dependent effect is observed on the CO_2_ sensing capacitance response at elevated temperatures. The sensing platform is highly selective to CO_2_ in comparison to N_2_ and O_2_. The overall variability in the N_2_ baseline and CO_2_ response recovery during cycling is found to be less than 1%. EMIM[TF_2_N] offers a novel solution for CO_2_ sensing due to its robust sensor performance metrics and stable electrochemical properties. The response of the developed low-power RTIL sensing platform are correlated to a commercial CO_2_ sensor to validate the efficency of the RTIL sensor calibration and accuracy of CO_2_ detection through regression analysis and Bland-Altman analysis. We observed a mean bias closer to zero and variability close to ~300 ppm on comparing the performances of both the CO_2_ sensors. The developed sensor platform is a promising step towards building a next generation rapid, low- power IOT based- microelectronic device for CO_2_ detection towards room occupancy monitoring applications.

## Method

### Reagents and materials

The RTIL candidate 1-ethyr-3-methylimidazolium [EMIM] and bis(trifuoromethane)sulfonimide [TF_2_N] (98% purity) was obtained from Sigma- Aldrich (St. Louis, MO, USA). High purity CO_2_ and O_2_ gas tanks were obtained from Airgas (Dallas, TX). The TI MSP-EXP430G2 MSP430G2 LaunchPad Development kit was obtained from Texas Instruments Inc. (Dallas, TX, USA). Operational amplifier LM358 and voltage regulators AP1117 were purchased from Digi-Key (Thief River Falls, MN, USA).

### Electrode design and material

A gold interdigitated electrode design (IDE) was utilized for CO_2_ and relative humidity sensing. Each digit of the IDE is 0.8 mm with digit separation of 0.2 mm and digit widths of 0.2 mm. The IDEs were custom designed and ordered from PCB Universe (Vancouver, WA, USA).

### CO_2_ sensing experimental setup

A custom-made environmental test system was built consisting of a gas mixer where the RTIL coated IDE’s were exposed to varying CO_2_ concentrations, humidity, and temperature variations. A nitrogen chamber (850-LCM/SP) from Pas-Labs, Inc was used to enclose the testing chamber from outer uncontrolled environmental conditions. A small galvanized box was built to contain the test RTIL-sensor with electrical connections to a potentiostat (Gamry Instruments) interfaced with a Torrey Pines Scientific, Inc. HS60 digital hotplate to control the temperature of the RTIL coated IDE’s. The smaller test chamber has inlets to provide CO_2_ and humidity to interact with the RTIL coated IDE’s for testing the sensor response. An Environics, Inc. 4000 series gas mixing system delivered the required CO_2_ concentrations for testing. This gas mixing system uses a 5% CO_2_ UPC grade cylinder combined with house N_2_ at greater than 95% purity for baseline measurements and for CO_2_ gas flow modulation. A commercial CO_2_ gas sensor- Vaisala GMW95RD CO_2_ monitor was placed in the nitrogen chamber to record the true measure of the CO_2_ concentration being provided to the test RTIL sensor.

### Experimental procedure to record the CO_2_ sensing response

The IDE’s were first soldered for easier interfacing with the Gamry; they were then cleaned with acetone, isopropyl alcohol, and dried with N_2_. The IDE’s were drop-coated with 1 μL of the RTIL to test the CO_2_ sensing response. The IDE’s were placed inside the small test chamber and then subjected to a heat treatment at 155 °C for 90 minutes in a pure dry N_2_ environment with 0% humidity to allow the RTIL to release any impurities. Following this, the IDE’s were cooled down to the test temperature in same environment. For baseline recording, EIS and CA measurements were performed in triplicate in an N_2_ environment with test humidity conditions maintained before introducing CO_2_ into the chamber. The N_2_ flow was then shut off and the test CO_2_ concentration was allowed to flow, while maintaining the test humidity conditions, into the smaller test chamber for 10 minutes for response testing. EIS and CA were then performed in triplicate measures to record the CO_2_ sensing response. For EIS, an AC perturbation voltage of 100 mV_rms_ was applied with a DC bias of 2.8 V is applied for recording the sensor response. The AC voltage was varied from 1 Hz to 10 KHz to record the double layer dynamics. For CA, a DC bias of 2.8 V was applied for a duration of 1-minute to record the steady-state current of the system in response to the test conditions of the chamber. Repeatability experiments were performed by incubating N_2_ and test CO_2_ concentration alternatingly over 20 cycles at test temperature and humidity conditions to record the variability in the CO_2_ sensing response. EIS and CA measurements were recorded in triplicate after each cycle. The cycling performance of the RTIL sensor was tested by incubating N_2_ and test CO_2_ concentration alternatingly over 5 cycles at test temperature and humidity conditions. The test sensor is heated to 155 °C for 60 minutes at 0% humidity after each CO_2_ cycle under a constant N_2_ flow to allow complete release of CO_2_ by the RTIL before capturing the CO_2_ response of the next cycle. EIS and CA measurements were recorded in triplicate after each cycle. All capacitance values have been extracted by fitting the impedance data to the Randle’s circuit using a circuit fit software ZView® by Scribner Associates.

### Fourier transform infra-red spectroscopy setup

The infrared spectra (IR) was collected using Nicolet iS-50 FTIR (Thermo Scientific Inc.) in Attenuated Total Reflectance mode. The tool comprised of deuterated triglycine sulfate (DTGS) detector and KBr window. The spectra were collected using Germanium crystal for 256 scans at a resolution of 4 cm^−1^ in the wavelength range 3500 cm^−1^ to 450 cm^−1^. Two samples were prepared by drop-coating 5 µL of RTIL on the electrode surface. One sample was used to obtain the reference RTIL spectrum and the other was saturated with 1000 ppm CO_2_ in the chamber by biasing it at the appropriate ECW voltage to capture the RTIL- CO_2_ interaction spectrum.

### CO_2_ detection on the low-power portable prototype

1 µL of RTIL was deposited onto the IDE’s and interfaced with the circuit. A Texas Instruments (TI) MSP 430 was used to apply a 20-millisecond square pulse at 3.3 V every 5 seconds which was stepped down to 2.8 V using a simple voltage divider according to the electrochemical window of the RTIL. The 2.8 V pulse was applied across the RTIL in series with a shunt resistor as a proxy for the current passing through the RTIL. The voltage drop across the resistor was amplified using a standard LM 358 operational amplifier with potentiometer adjustable gain. The amplified signal was received by the microcontroller for signal processing. Additionally, a 3.3 V supply to the MSP 430. An RGB LED was also added to provide an easy and interactive indication of carbon dioxide concentration. The time average of the current signal was calculated over the period of the applied pulse. The most recent 200 measurements were stored in an array and used to calculate a moving average of the signal to account for slight changes in the environment. Provisions were made such that this baseline would not move under the influence of large gusts of carbon dioxide (for example, a person’s breath) but only gradually due to environmental changes. The percent change between this baseline and the most recent raw measurement was converted into a parts per million carbon dioxide concentration. This measurement was found to be noisy and contained large semi-periodic spikes that were difficult to filter using traditional techniques without obstructing the signal itself. Therefore, a buffer was inserted, and a short algorithm was used to check if the most recent measurement was significant or not. For this, there was at most a 5 second delay before the most recent measurement could be seen on the MSP 430’s on board LCD screen.

### Statistical analyses

All the data is analyzed using OriginPro. SEM and RSD are calculated for the number of replicates or repeats used for experimentation and are mentioned in the results section. N = 3 replicates have been used for experimentation throughout the manuscript.

## Supplementary information

CO2 sensor operation demonstration.

Supplementary information.

## Data Availability

Data generated and analyzed during this study is available from the corresponding author on request.
