# Supplementary material for: CLIP: Carbon Dioxide testing suitable for Low power microelectronics and IOT interfaces using Room temperature Ionic Liquid Platform
Source: Sci Rep. 2020 Feb 13;10:2557. doi: 10.1038/s41598-020-59525-y (PMC7018756; doi:10.1038/s41598-020-59525-y)

**CLIP: Carbon Dioxide testing suitable for Low power microelectronics and IOT interfaces using Room temperature Ionic Liquid Platform**

Ashlesha Bhide^1^, Badrinath Jagannath^1^, Ambalika Tanak^1^, Richard Willis^2^, Shalini Prasad^1*^

^1^*Department of Biomedical Engineering, University of Texas at Dallas, 800 W Campbell Rd., Richardson, TX 75080*

^2^*Department of Electrical and Computer Engineering, University of Texas at Dallas, 800 W Campbell Rd., Richardson, TX 75080*

*Correspondence to [Shalini.Prasad@utdallas.edu](mailto:Shalini.Prasad@utdallas.edu)

**Supplementary information**

**Supplementary S1**. The electrode boundary conditions were applied to the working electrode and counter/reference electrode was ground/insulated. A potential of 10 mV was applied to the working electrode with a DC bias of 2.8V. Neumann’s boundary condition (n. J = 0) was applied to the electrolyte layer. The equations governing the simulated potential and current density are listed below-

Electrode: $J_{s}= {-\sigma}_{s}{\nabla\varphi}_{s} with {\nabla J}_{s}=\varphi_{s}$

Electrolyte: $J_{l}= {-\sigma}_{l}{\nabla\varphi}_{l} with {\nabla J}_{l}=\varphi_{l}$

Electrode- Electrolyte interface = $\varphi_{s}-\varphi_{l}=E_{eq}$

*J* represents the current density vector (A/m^2^), which is dependent on *σ*, the conductivity (S/m), and φ, the electric potential (V). The index *s* denotes the electrode domains, while the *l* subscript denotes the electrolyte domain. *E_eq_* is the potential difference at the electrode–electrolyte interface.

The electric field ‘E’ can be obtained from the following relation and the gradient of the scalar potential V as given below –

$$D= \varepsilon_{0}\varepsilon_{r}E$$

$$E= -\nabla V$$

D is the displacement current, ε_0_ is the permittivity of free space and ε_r_ is the relative permittivity of the material/electrolyte used.

Figure S1. COMSOL simulations for a) Electrode potential distribution b) Electrode current density


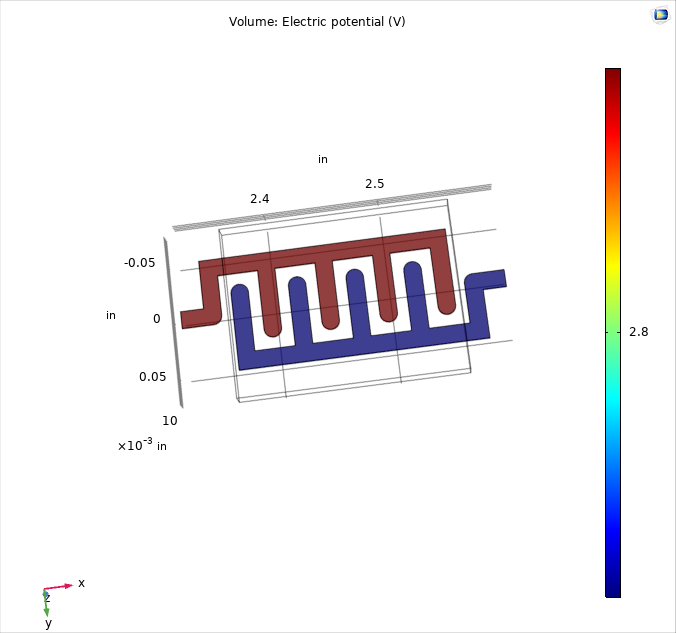


(a)


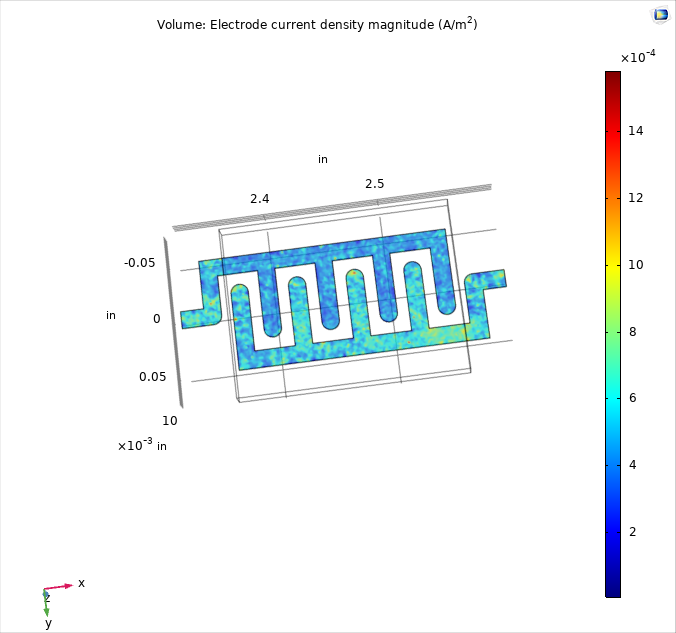


(b)

**Supplementary S2.** Dominant capacitive effects are observed in the regime 100- 1000Hz wherein a capacitive phase is observed at 100Hz. A circuit fit model is used to extract the double layer capacitance ‘C_dl_’ values at 100Hz. N_2_ baseline and CO_2_ response bode plots are shown in the figures below.


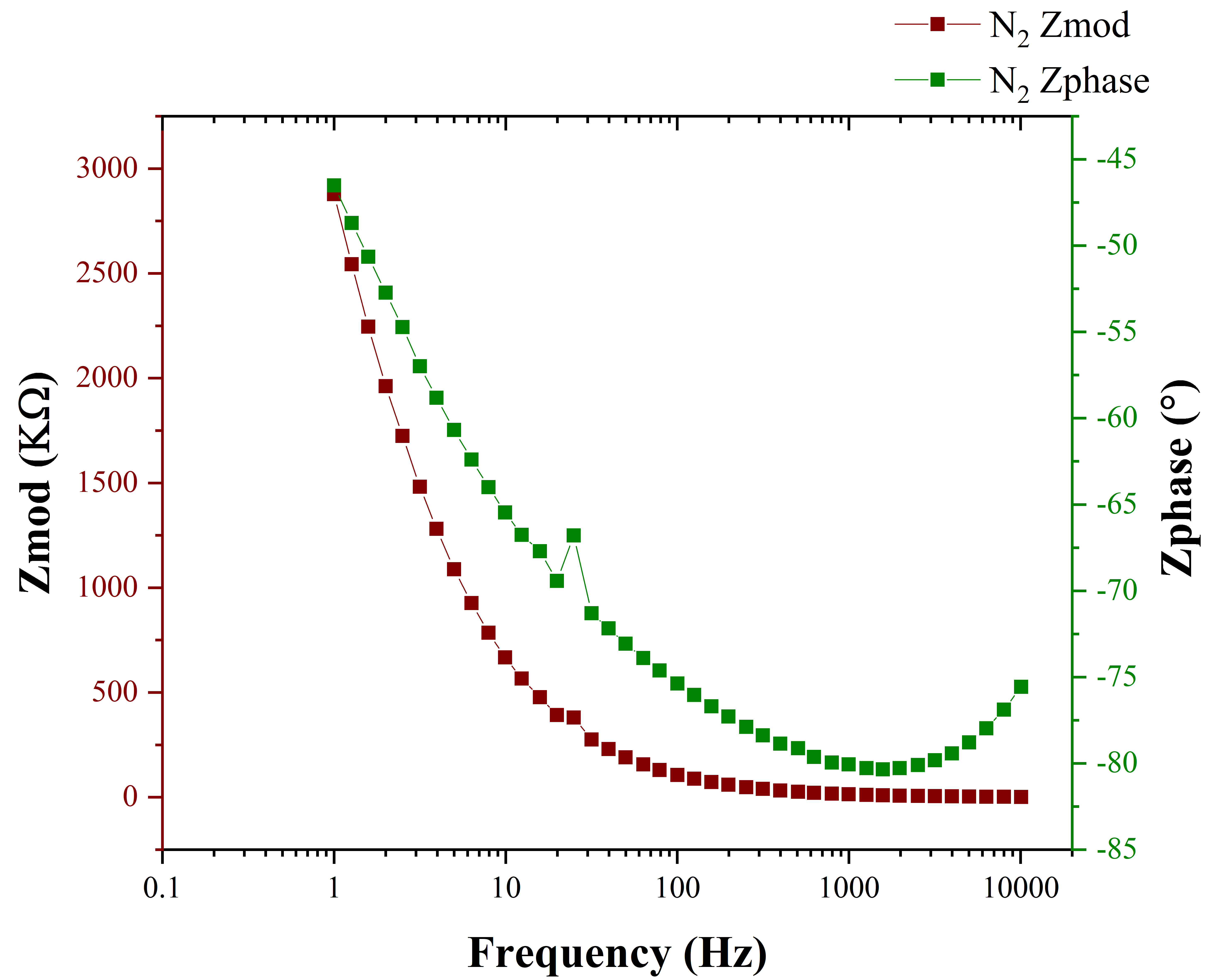


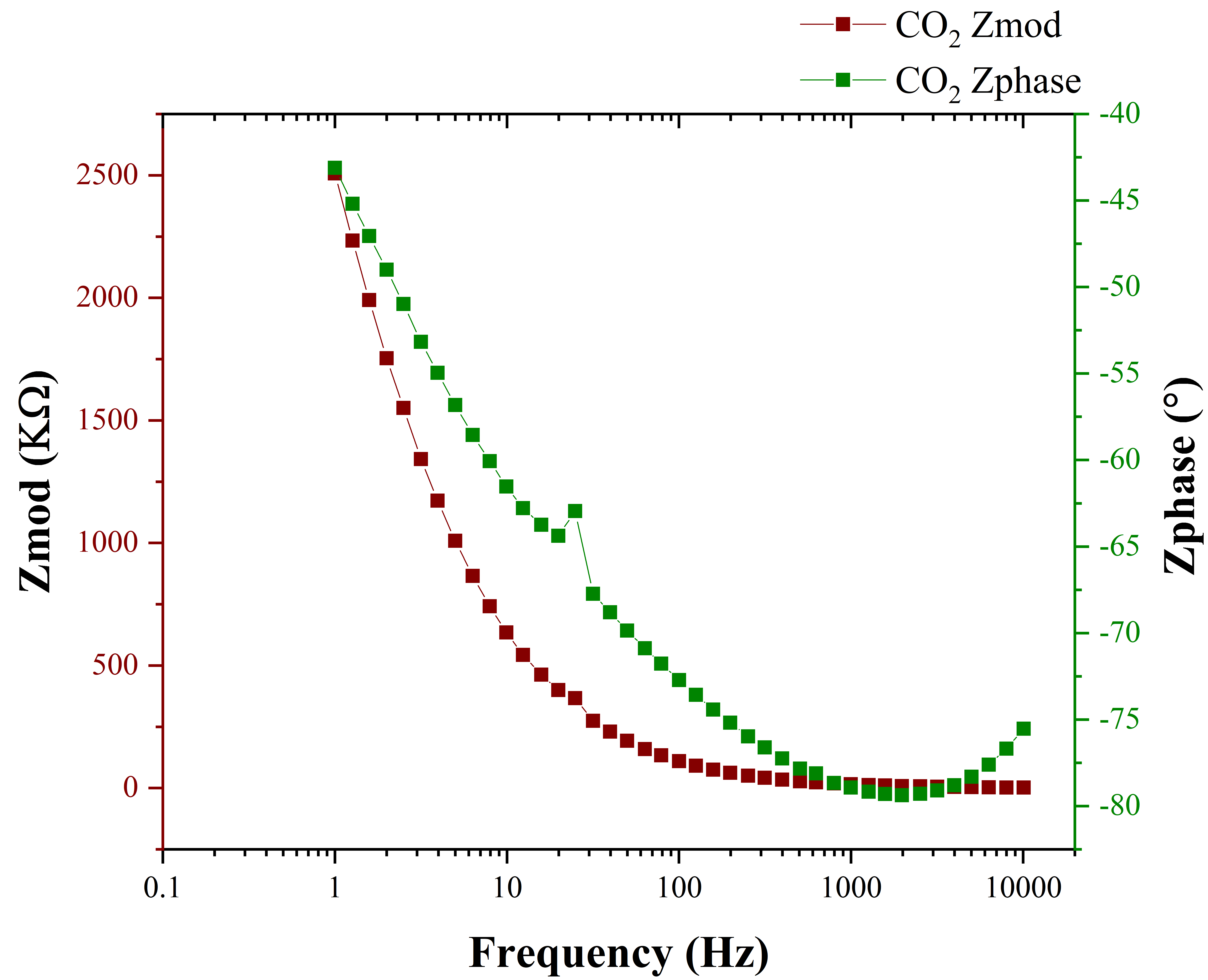


**Supplementary S3.**

Repeatability of the sensor at elevated conditions -65ºC 65%RH


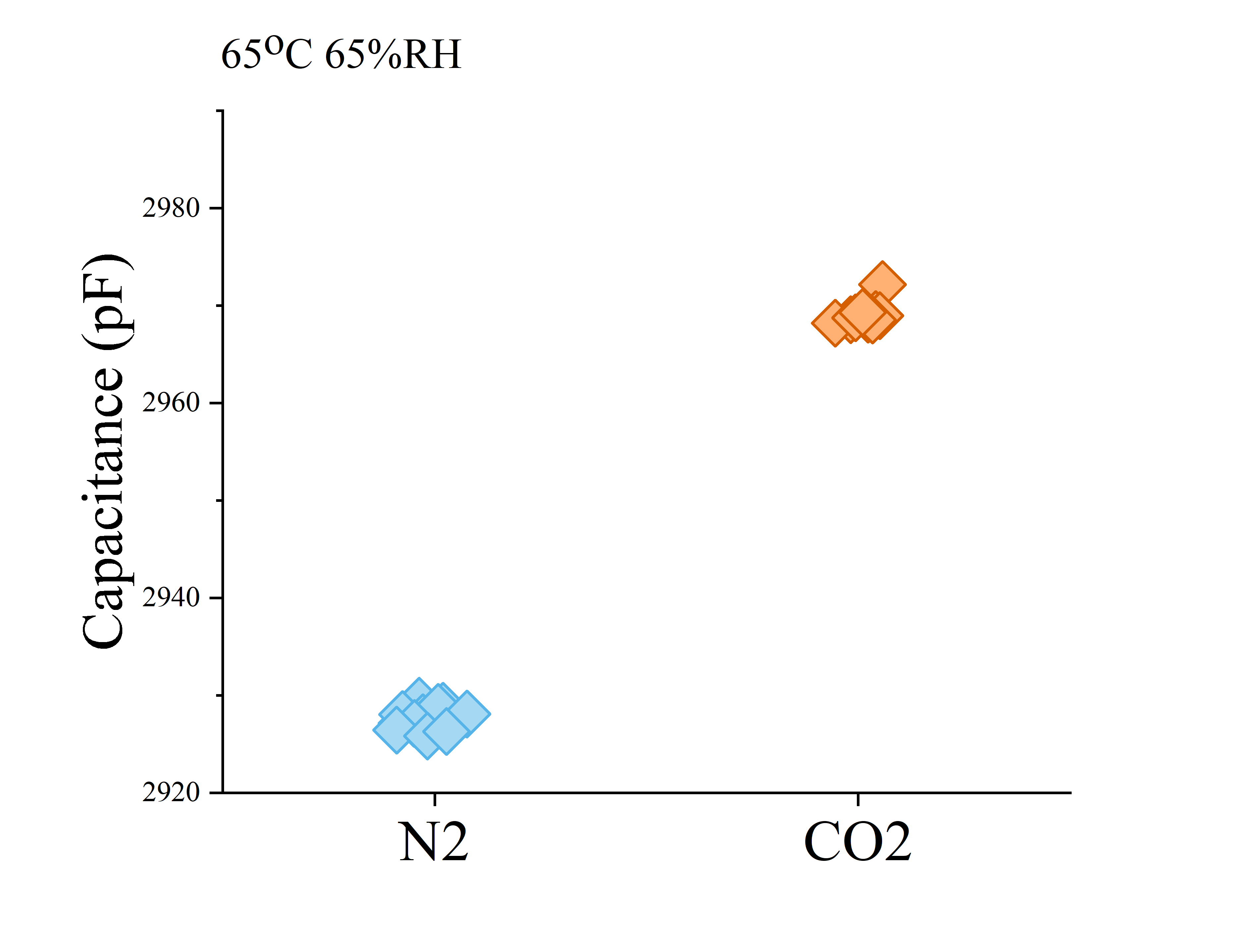


Continuous cycling performance of the sensor at elevated conditions -65ºC 65%RH


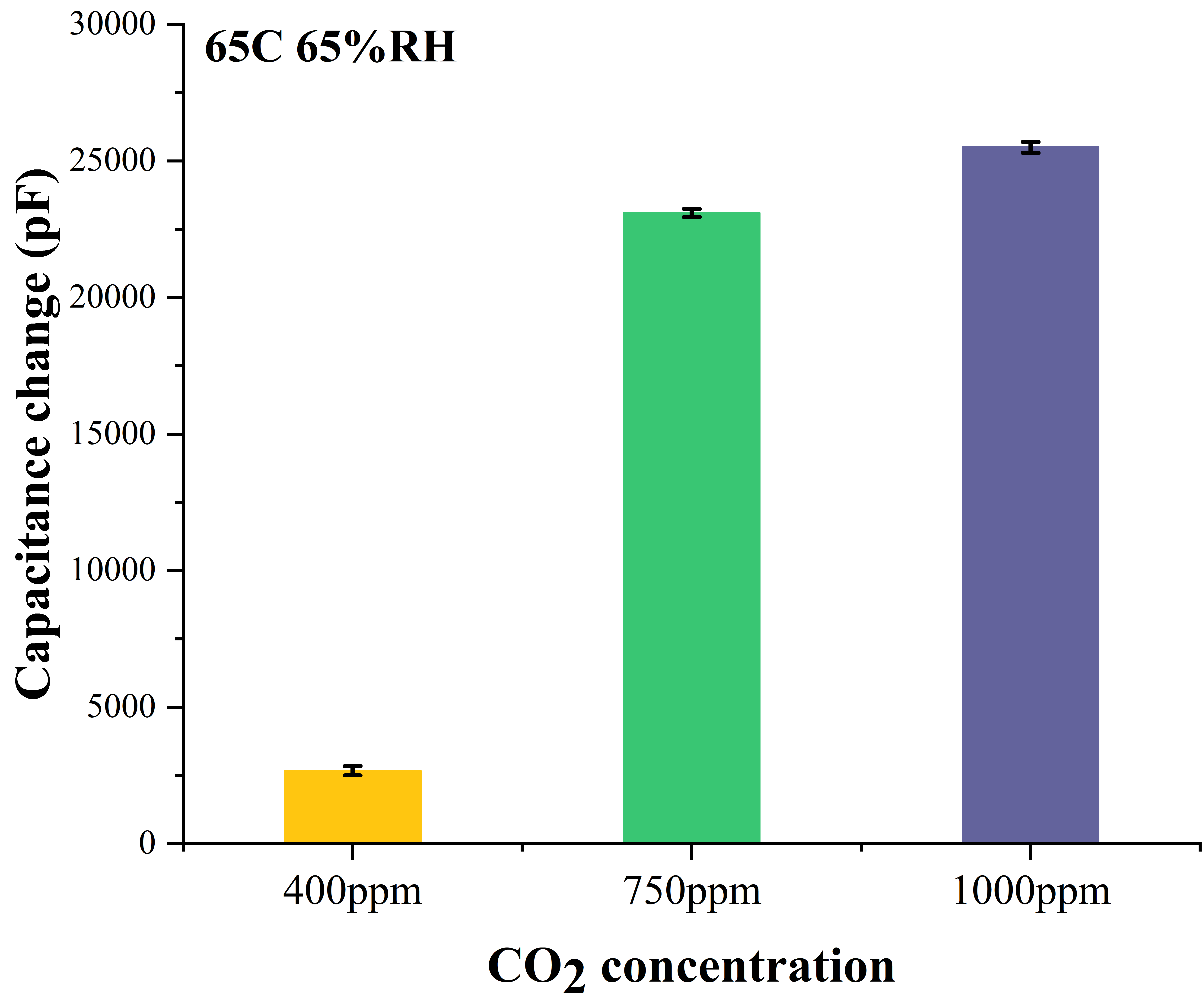

Supplement: Supplementary file 2 — Supplementary information. [file 41598_2020_59525_MOESM2_ESM.docx]
